# The Effect of *Rosa spinosissima* Fruits Extract on Lactic Acid Bacteria Growth and Other Yoghurt Parameters

**DOI:** 10.3390/foods9091167

**Published:** 2020-08-24

**Authors:** Marek Szołtysik, Alicja Z. Kucharska, Anna Sokół-Łętowska, Anna Dąbrowska, Łukasz Bobak, Józefa Chrzanowska

**Affiliations:** 1 Department of Functional Food Products Development, Wrocław University of Environmental and Life Sciences, Chełmońskiego Str. 37, 51-640 Wrocław, Poland; anna.dabrowska@upwr.edu.pl (A.D.); lukasz.bobak@upwr.edu.pl (Ł.B.); jozefa.chrzanowska@upwr.edu.pl (J.C.); 2 Department of Fruit, Vegetable and Plant Nutraceutical Technology, Wrocław University of Environmental and Life Sciences, Chełmońskiego Str. 37, 51-640 Wrocław, Poland; alicja.kucharska@upwr.edu.pl (A.Z.K.); anna.sokol-letowska@upwr.edu.pl (A.S.-Ł.)

**Keywords:** *Rosa spinosissima* fruits, phenolic compounds, extract, yoghurt, antioxidant properties

## Abstract

The aim of the study was to evaluate the effect of purified extract from *Rosa spinosissima* fruits on the quality characteristics and antioxidant properties of yoghurt. The extract, added to yoghurt at a concentration of 0.1% and 0.2%, contained high quantities of phenolic compounds and exhibited high antioxidant activity due to the presence of anthocyanins flavan-3-ols, flavonols and ellagitannins. Yoghurt physicochemical properties, microbiology and antioxidant properties were evaluated after 1, 7 and 14 days of storage at a temperature of 4 °C. The data revealed a positive influence of rose preparation on yoghurt’s microflora and on its other properties. The highest count of traditional yoghurt microflora was observed in samples with 0.2% of extract. Its addition had a positive effect on the yoghurts’ color, giving them a characteristic pink color of an intensity dependent on additive concentration. It also significantly affected the yoghurts’ antioxidant properties, which were stable during storage, as well as the content of the introduced phenolic compounds.

## 1. Introduction

Nowadays, more attention is given by consumers to functional food, which is enriched in bioactive substances revealing a wide range of health benefits. These substances of diverse chemical structure and function have demonstrated positive effects, especially in the prevention and treatment of diet-related diseases, including cardiovascular disorders, stroke, renal diseases, osteoporosis and diabetes [1]. Among such bioactive substances are, originating from plants, a wide variety of compounds exhibiting antioxidant properties. Antioxidants play an important role in organism protection against damage induced by reactive oxygen species such as O^2−^, OH^−^ and H_2_O_2_ under conditions of elevated oxidative stress [2]. In food, antioxidants retard the formation of toxic oxidation products, control rancidity development, maintain nutritional quality and extend the shelf-life of products [3]. A High content of these compounds is found in the plants of the *Rosaceae* family that are used by both food and pharmaceutical industries [4]. One of the most widespread members of this family is the genus *Rosa*, comprising of about 190 species, which are widely distributed throughout the northern hemisphere [5]. The fruits of roses are especially rich sources of antioxidants, which is attributed to the fact that they contain an abundance of compounds including phenols, carotenoids, lycopene, vitamin C, tocopherol, pectin, sugar, organic acids, amino acids and essential oils [6]. Among these substances, phenolic compounds, grouped into the four main classes of flavonoids, phenolic acids, tannins and stilbenes, were shown in many studies to be very effective antioxidants in vitro that might contribute significantly to protective effects in vivo [7]. However, depending on species and variety, growing conditions or the maturity stage of rose hips, fruit composition and concentration of these substances may differ significantly [4,5,6,8]. According to various researchers, the quantity of these compounds were in ripe rose hips between 176–9600 mg gallic acid equivalents per 100 g [6,9].

*Rosa spinosissima* (synonym *Rosa pimpinellifolia*), used as a raw material in our research, differs from other species of the Rosa genus because of the very dark color of its fruits, which are even black, which is attributed to the exceptionally high content of anthocyanins, the concentration of which Babis and Kucharska [10] determined to be in the range of 482–544 mg/100 g.

In comparison with fresh fruits, the concentrated fruit extracts are several times richer in phenolic compounds. Therefore, the aim of our study was to evaluate *Rosa spinosissima* fruit’s dry extract as a functional additive in yoghurt production and to conduct an analysis of how the additive influences the product characteristics, especially its antioxidant potential. Extracts, as well as the fruits of the *Rosa* genus, have not been used as additives in yoghurt production before. Preferentially, pomace, pulps or water extracts of other plants are used in this kind of application, whereas dry polyphenolic extracts are used rarely [11,12,13]. Yoghurt, due to its high nutritional value and organoleptic properties, is an attractive product, which is an important component of daily diets [14]. Besides its high content of valuable components such as proteins, lipids, lactose and minerals, it contains lactic acid bacteria (LAB) responsible for the fermentation process, which have a significant positive impact on yoghurt nutritional properties. Yoghurt consumption is increasing all over the world, which is caused also by the introduction to it of different antioxidant additives that increase its health effects [11].

## 2. Materials and Methods

### 2.1. Reagents

Methanol, acetic acid, 1,1-Diphenyl-2-picrylhydrazyl radical (DPPH), 2,2′-azinobis(3-ethylbenzothiazoline-6-sulfonic acid) (ABTS), 2,4,6-tri(2-pyridyl)-s-triazine (TPTZ), FeCl_3_ and 6-hydroxy-2,5,7,8-tetramethylchroman-2-carboxylic acid (Trolox) were purchased from Sigma-Aldrich (Steinheim, Germany). Cyanidin 3-O-glucoside (Cy glc), Ellagic acid (EA), (+)-Catechin (Cat), Quercetin 3-O-glucoside (Q glc) were purchased from Extrasynthese (Lyon Nord, France). MRS agar and M17 agar were obtained from Merck (Germany). Acetonitryl for liquid chromatography–mass spectrometry and other chemicals were purchased from POCh (Gliwice, Poland).

### 2.2. Preparation of Polyphenol Extract from Rosa Spinosissima

Frozen ripe fruits of *R. spinosissima* (1 kg) were shredded and heated for 5 min at 95 °C using a Thermomix (Vorwerk, Wuppertal, Germany). The pulp was subsequently cooled down to 40 °C and depectinized at 50 °C for 2 h by adding 0.5 mL of Panzym Be XXL (Begerow GmbH & Co., Darmstadt, Germany) per 1 kg. After depectinization, the pulp was pressed in a Zodiak laboratory hydraulic press (SRSE, Warsaw, Poland). The pressed juice was filtered and run through an Amberlite XAD-16 resin column (Rohm and Haas, Chauny Cedex, France). Impurities were washed off with distilled water, while phenolic compounds were eluted with 80% ethanol. The eluate was concentrated under a vacuum at 40 °C. The solvent was evaporated using a Rotavapor (Unipan, Warsaw, Poland) and the extract was lyophilized (Alpha 1–4 LSC, Christ, Germany), which resulted in obtaining the dry extract used as an additive in yoghurt production.

### 2.3. Yoghurt Preparation

Yoghurt was made from commercial pasteurized milk of 3.2% fat content. The dry matter of milk was increased by 2% with the use of skimmed milk powder and heat treated at a temperature of 90 °C for 10 min. *R. spinosissima* dry extract was added to milk before inoculation at concentrations 0.1% and 0.2%. Control yoghurt was made only with the addition of skimmed milk powder. Yoghurt culture composed of *Streptococcus thermophilus* and *Lactobacillus bulgaricus* (Yo-flex, CHR Hansen) was added to milk at a concentration of 2% (*v*/*v*). Incubation was carried out at 44–45 °C until a pH of 4.6–4.7 was reached (ca. 4.5 h). Yoghurt samples were analyzed on the first day after production and after 7 and 14 days of storage at a temperature of 4 °C. The yoghurt cups of each sample group were used for physicochemical, microbial and antioxidant activity determinations.

### 2.4. Physicochemical Analysis of Yoghurt

The pH of samples was measured using an InoLab pH-meter. Titratable acidity was determined according to the Soxhlet-Henkel method and expressed in SH degrees defined as the amount of 0.25 M NaOH used for the titration of 100 mL of yoghurt in the presence of an indicator (phenolphthalein).

### 2.5. Color Measurement

The surface color of yoghurt samples was measured by Minolta Chroma Meter CR-400 (Konica Minolta, Japan) and expressed in L*/(lightness; 100 = white, 0 = black), a*/(redness; ±, red; green) and b*/(yellowness; ±, yellow; blue) values. Calibration readings of the reference were carried out using a white plate.

### 2.6. Rheological Properties

Yoghurt samples were removed from refrigerated storage and equilibrated at room temperature for 0.5 h prior to rheology measurements. The rheological characterization of the yoghurt samples was performed in a Haake RheoStress 6000 rotational rheometer with a Haake A10 thermostatic bath and a UTM Controller (Thermo Electron GmbH, Karlsruhe, Germany). The measurement was made at a constant temperature (20 °C) using a cone/plate (cone C60/1° Ti L no. 222-1868/stainless steel plate TMP60 no. 222-1891) geometry system with a gap of 1 mm for all samples. A sample of yoghurt (1 mL) each time was applied to the surface of the plate. All viscosity measurements were expressed in centipoises seconds (cPs), performed in triplicate and were determined three times for each sample with a ramp shear rate in the range of 0 to 1000 s^−1^ over 3 min. Apparent viscosity was determined at a shear rate of 100 s^−1^.

### 2.7. Microbiological Analysis

Starter bacteria count was carried out by using selected media. *Streptococcus thermophilus* was counted on M-17 (Merck) and *Lactobacillus delbrueckii subsp. bulgaricus* on MRS agar (Merck). Plates with streptococci and lactobacilli were incubated at 37 °C for 48 and 72 h, respectively according to ISO 7889/IDF 117 (2003).

### 2.8. The Total Phenolic Compound (TPC) Content Determination

The total phenolic compound (TPC) content was estimated using the Folin–Ciocalteu (FC) reagent method as described by Singleton et al. [15] The reaction mixture was prepared by mixing 500 μL aqueous extract, 975 μL 2% Na_2_CO_3_ and 25 μL FC reagent dissolved in deionized water. After 1 h incubation at room temperature (25 °C), the absorbance was measured against a blank at 765 nm using a UV-2401 PC spectrophotometer (Shimadzu, Kyoto, Japan). Gallic acid was used as a standard and total phenolics were expressed as mg gallic acid equivalent (GAE) per 100 g of fresh weight.

### 2.9. Antioxidant Activity

The DPPH radical scavenging activity of samples was determined according to the method of Yen and Chen [16]. The absorbance was measured after 10 min at 517 nm. The ABTS method described by Re et al. [17] was used for assay. The absorbance was measured after 6 min at 734 nm. Ferric-reducing antioxidant power (FRAP) was measured according to Benzie and Strain [18]. The absorbance was measured after 10 min at 593 nm. All determinations were performed in triplicate using a microplate reader Synergy H1 (BioTek, Winooski, VT, USA). The results of all antioxidant activity determinations were expressed in mmol Trolox equivalent (TE) per 100 g fresh weight. For the quantification of these activities, a calibration curve in the range 0.01–5.00 mmol of Trolox was used.

### 2.10. Extraction of Phenolic Compounds from Yoghurt

Extraction of phenolic compounds from yoghurt was conducted with acidified methanol as described by Trigueros et al. [19]

### 2.11. Quantification of Phenolic Compounds by the HPLC-PDA Method

The HPLC-PDA method was previously described by Kucharska et al. [20] The quantification analysis was performed using a Dionex (Germering, Germany) system, equipped with the diode array detector model Ultimate 3000 quaternary pump LPG-3400A, autosampler EWPS-3000SI, thermostated column compartment TCC-3000SD, and controlled by Chromeleon v.7.2.9 software (Thermo Scientific Dionex, Sunnyvale, CA, USA). The Cadenza Imtakt column C5-C18 (75 × 4.6 mm, 5 µm) was used. The mobile phase was composed of solvent A (4.5% aq. formic acid, *v*/*v*) and solvent B (100% acetonitrile). The elution system was as follows: 0–1 min 5% B in A, 1–20 min 25% B in A, 20–21 min 100% B, 21–26 min 100% B, 26–27 min 5% B in A. The flow rate of the mobile phase was 1.0 mL/min and the injection volume was 20 µL. The column was operated at 30 °C. Anthocyanins were detected at 520 nm, flavonols at 360 nm, flavan-3-ols at 280 nm and ellagitannins at 254 nm. Anthocyanins were expressed as cyanidin 3-O-glucoside, flavonols as quercetin 3-O-glucoside, flavan-3-ols as (+)-catechin and ellagitannins as ellagic acid (Figure S1 and Table S1 in Supplementary Materials). The results (mean values from 3 experiments ± standard deviation; SD) were expressed as mg per 1 L (for yoghurt) and mg per 100 g (for fruits and purified rose extract).

### 2.12. Statistical Analyses

Graphs for analysis, mean values ($\bar{X}$) and standard deviation (SD) were calculated in excel spreadsheet (Microsoft Office version 2016). Data were analyzed by one-way ANOVA using Statistica 13.1 software (TIBCO, Palo Alto, CA, USA). Differences between means were determined by Duncan’s test (*p* < 0.05). All experiments were carried out in triplicate. Letters ^a,b,c,d,etc.^ show the homogeneous groups.

## 3. Results and Discussion

Fruits of *R. spinosissima* are known to be a rich source of phenolic compounds [21]. In our research, we confirmed the high content of total phenolic compounds and antioxidant activities in fruit and in purified dry preparation from fruits of this species (Table 1). TPC content in fruit reached the level of 2643.43 ± 56.57 mg GAE/100 g and showed antioxidant activity determined by the use of three assays: DPPH, FRAP and ABTS at the levels of 11.01 ± 0.74; 18.33 ± 0.71 and 58.87 ± 1.83 mmol TE/100 g, respectively. Extract, purified on Amberlite resin after stabilization by freeze-drying, resulted in dry preparation obtained with a yield of 0.6%. Compared to fruit, it exhibited an about. 18 times higher content of TPC and revealed a significantly higher antioxidant potential. Its antioxidant activity, determined by the use of the abovementioned assays, were at the levels of 144.36 ± 1.87, 328.62 ± 2.86 and 517.28 ± 3.21 mmol TE/100 g, respectively.

Compared with other rose fruit extracts, it exhibited a higher content of TPC. Koczka et al. [4], who also evaluated these compounds in extracts from the hips of selected *Rosa* species (*R. spinosissima*, *R. canina*, *R. rugosa* and *R. gallica*), discovered that TPC content ranged from 255.9 mg to 766.0 mg GAE/100 g DW (dry weight). These authors also confirmed that *R. spinosissima* had the highest total phenolic content and antioxidant capacity, significantly higher than the other investigated *Rosa* species. But Fattahi et al. [9] in their research showed a similar level of total phenolic compounds in *R. spinosissima* and *R. canina* fruits, while Murathan et al. [22], during an analysis of four different rosehip species growing naturally in Eastern Anatolia in Turkey, detected in the analyzed fruits the lowest content of phenolic compounds (1058 mg/100 g) but with the highest contribution of anthocyanins.

Many authors emphasize the correlation between the antioxidant activity of rose fruits and their total polyphenolic content. In numerous studies, it has been shown that antioxidant activity of plant extracts is correlated with total phenolics rather than with individual phenolic compounds. However, different phenols develop different activities depending on their chemical structure [5,22]. Some researchers point out that the activity may also be influenced by the presence of some other compounds with a high antioxidant capacity, such as vitamin C, pigments and tocopherols [8].

**Figure 1:**
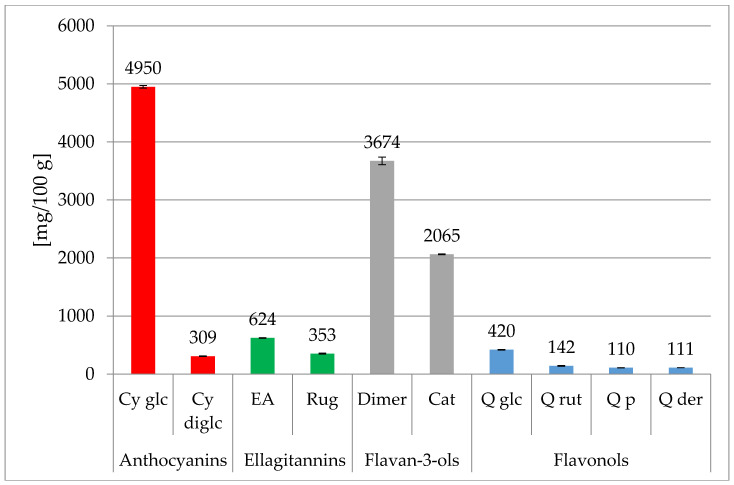
Content of phenolic compounds (Cy glc: cyanidin 3-O-glucoside; Cy diglc: cyanidin 3,5-O -diglucoside; Dimer: procyanidin dimer; Cat: (+)-catechin; EA: ellagic acid; Rug: rugosin; Q glc: Quercetin 3-O-glucoside; Q rut: Quercetin 3-O-rutinoside; Q p: Quercetin 3-O-pentoside; Q der: Quercetin derivative in the purified dry extract from fruits of *Rosa spinosissima*). The bars represent mean values of three experiments ± SD.

The content of individual phenolic compounds in *R. spinosissima* purified dry extract was analyzed with the use of high pressure liquid chromatography HPLC-PDA (Figure 1; Figure S1 and Table S1 in Supplementary Materials). In the extract, 10 phenolic compounds with the summary value of 12,759.1 mg/100 g were identified, among which anthocyanins were determined in the amount of 5259.2 mg/100 g (i.e., 41.22%). In that group, an exceptionally high amount of cyanidin-3-O-glucoside was determined: 4950.0 mg/100g (38.80% of all phenolic compounds). Moreover, Guimaraes et al. [23] reported that the major anthocyanin in *R. canina* fruits was cyanidin-3-O-glucoside. The second in amount of compound among anthocyanins identified in the obtained *R. spinosissima* preparation was cyanidin 3.5-O-diglucoside: 309.2/100 g. Many authors discovered that anthocyanins were the predominate group of phenolic compounds in fruits of this species [4,10]. However, in our research, the highest content of 5738.2 mg/100 g, covering 44.97% of all phenolic compounds in dry preparation, was determined for flavan-3-ols and within the group of procyanidin dimers: 3673.6 mg/100 g and catechins: 2064.6 mg/100 g. Among flavonols, four compounds were identified with the highest content of quercetin 3-O-glucoside (4203 mg/kg). Ellagitannins discovered in the preparation were represented by ellagic acid and rugosin at the concentrations of 624.3 and 352.9 mg/100 g, respectively.

The content and amount of individual polyphenolic compounds in rose fruits of different species is also diverse. Stanila et al. [24], using HPLC-ESI-MS and diode-array detection, found in raw extracts of *Rosa canina var. lutetiana f. flexibilis* 19 compounds belonging to the anthocyanins, flavonol glycosides and phenolic acids. Whereas Demir et al. [5], in extracts of *Rosa canina*, *Rosa dumalis*, *Rosa gallica, Rosa dumalis subsp.boissieri* and *Rosa hirtissima* fruits, detected, by the RP-HPLC-DAD method, the presence of 18 different phenolic compounds among which gallic acid, catechin, procyanidin-B2 and hydroxycinnamic acid derivatives (chlorogenic, t-caffeic, p-coumaric, ferulic and sinapic acids) were principal for all rose hip species. Bhave et al. [8], using UHPLCeHRMS, determined in the fruits of roses belonging to 71 different genotypes (both pure species and hybrids) 12 free and conjugated polyphenolic/flavonoid compounds and discovered that *R. spinosissima* contained significantly higher levels of methylgallate hexoside than other samples.

The dry preparation of *R. spinosissima* fruits was applied at a concentration of 0.1% and 0.2% for yoghurt production. The pH and total acidity changes of the obtained products are shown in Table 2. The pH of all analyzed samples on the first day after production of control yoghurt and yoghurts with the addition of *R. spinosissima* extract were at the similar level of 4.38–4.41. Extension of storage time resulted in lowering the pH values in all analyzed samples. However, all yoghurts were characterized by similar acidification dynamics and after 14 days of storage, the determined pH values were 4.31–4.33.

The total acidity of analyzed yoghurts after one day of storage was in the range of 42.1 to 44.5 °SH. During further storage, that parameter increased in all samples up to 49.6–50.7 °SH. The applied statistical analysis showed that in the produced yoghurts, the storage period influenced the acidity changes significantly (*p* < 0.05), while no such influence was observed for applied variants.

The obtained results of yoghurt acidity show changes typical for that kind of products coming from the enzymatic degradation of milk components conducted by starter microflora. Similar trends in acidity changes during cool storage of yoghurts were observed by other authors [25,26], who applied as yoghurt additives dry plant preparation: wine grape pomace powder and papaya peel powder, respectively.

Yoghurt color is one of the basic parameters to which consumers pay their attention, as its reflects yoghurt quality and attractiveness [27]. The addition of analyzed *R. spinosissima* preparation gave the product a pink color similar to the color of commercial strawberry yoghurts. According to the results obtained for the color parameter analysis (Table 3), significant changes (*p* < 0.05) were correlated with the amount of applied additive. On the first day after production, their L* parameter, reflecting the brightness of the yoghurts, showed the statistically significant differences between control and other analyzed samples. In the control sample, it reached the value of 86.19 ± 2.89, while in samples with the *R. spinosissima* preparation, it was lower at 72.77 ± 1.86 and 69.18 ± 0.08 for samples with 0.1% and 0.2% addition of analyzed preparation, respectively. On 14 day of yoghurt storage, the parameter L* (lightness) did not undergo any significant changes. The statistically significant differences were also observed for the a* parameter (redness). In the control, the parameter took negative values, which indicates the higher contribution of the green color in the sample. This parameter decreased during yoghurt storage from the value of −2.34 ± 0.20 to −2.48 ± 0.05. The b* (yellowness) parameter however underwent only insignificant changes in control (from the value of 6.21 ± 0.50 to 6.23 ± 0.30). In yoghurts with addition of rose preparation, the a* parameter took positive values with the higher contribution of red color. On the first day after production, its value was 8.71 ± 0.68 and 13.3 ± 0.06 in samples with 0.1% and 0.2% of additive, respectively. After 14 days of storage, parameter a* was lowering and this decrease was higher in samples with a higher dose of *R. spinosissima* preparation. In contrast to control, parameter b* got negative values in experimental yoghurt samples, which points to the higher contribution of the blue color in these products. The refrigeration storage influenced on the decrease of this parameter from −0.44 ± 0.01 to −0.31 ± 0.05 and from −0.62 ± 0.06 to −0.54 ± 0.10 in yoghurts enriched with 0.1% and 0.2% of additive. The color changes, like lightening, in food products, as it was pointed by Ścibisz et al. [12], may come from the lower stability of anthocyanins.

**Figure 2:**
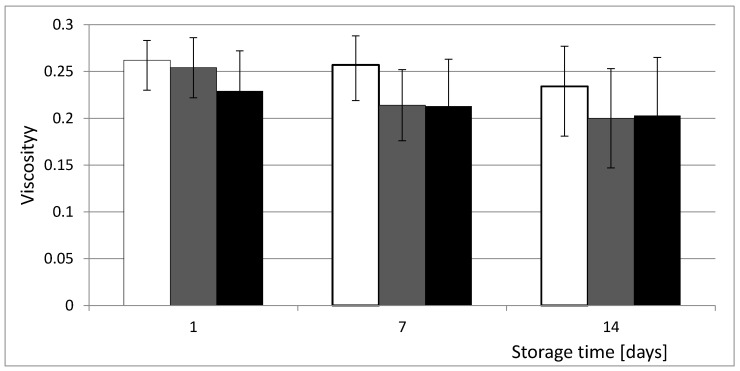
Viscosity of yoghurts supplemented with *Rosa spinosissima* fruits extract during 14 days of storage. □—Control, ■—0.1% and ■—0.2% addition of *Rosa spinosissima* fruits extract.

Yoghurts are characterized by gel structure developing during their production. The rheological properties are revealed during the fermentation process and are influenced by the applied technology, activity of starter cultures and the additives used during production [28]. The results of yoghurt rheological analysis are in Figure 2. The viscosity of yoghurts with the addition of rose preparation, especially with the dose of 0.2%, took lower values in comparison to control samples on the first day (0.229 ± 0.043 vs. 0.262 ± 0.021 cPs in control) and during the entire storage period. During this time, the parameter showed a decreasing tendency towards what was particularly observed between 7th and the 14th day of storage. On the last day of product analysis, it took the values of 0.234 ± 0.043 cPs in control and 0.200 ± 0.050 and 0.203 ± 0.062 cPs in samples with 0.1% and 0.2% of preparation, respectively. However, the observed differences between experimental and control yoghurts appeared to be statistically insignificant. Many authors have investigated the influence of different additives on the rheological parameters of yoghurts. Dabija et al. [29], who evaluated yoghurts enriched with different herb extracts, also showed their lower viscosity values in comparison with the control sample. While El Said et al. [13], evaluating yoghurts fortified with pomegranate peel extract, concluded that the increase of the additive concentration negatively influenced the viscosity, which was explained by its impact on the aggregation of the casein network in yoghurts via electrostatic interaction, and in the resistance of the yoghurt matrix to flow. However it is noteworthy that these authors fortified yoghurts with a significantly higher concentration of additive (5–35%) than was used in our studies.

The microorganism count of analyzed yoghurts, showed in Table 4, was maintained over 8 log10 CFU/mL, however the number of cocci was higher than lactobacilli. The highest count of both microbial groups was determined in yoghurts with the addition of 0.2% of rose preparation, the lowest in control samples. One day after production, in this experimental sample, the population of cocci and lactobacilli reached the level of 8.98 ± 0.08 and 8.31 ± 0.02 log10 CFU/g, while in control it was 8.45 ± 0.04 and 8.13 ± 0.12 log10 CFU/g, respectively. With storage time, the count of *Str. thermophilus*, as well as *Lb. bulgaricus*, was lowering. The performed statistical analysis showed that the yoghurt variant and the time of storage were statistically significant (*p* < 0.05) for the yoghurt microflora growth. The decrease in the LAB count in yoghurts with the addition of phenolic compounds was reported by many authors, which is explained by the lowering of pH and the increase in acidity of the product. Discreet changes in yoghurt microflora after six weeks of storage were also noted by Jaziri et al. [30] who applied as an additive green and black tea leaves rich in phenolic compounds, especially in cathechins. Other authors observed that plant substances such as moringa, persimmon and white mulberry leaf powders or cardamom increased the counts of traditional yoghurt microflora and probiotic bacteria such as *Bifidobacterium* [31,32,33].

The application of rose polyphenolic preparation to yoghurts increased their antioxidant potential (Table 5), which was positively correlated with the preparation dose and was higher in samples with a 0.2% addition of *R. spinossima* preparation. It is particularly noticeable in tests analyzing the free radicals scavenging activity, DPPH and in ferric-reducing antioxidant power, which showed that the activities in yoghurt samples with the addition of 0.1% of *R. spinosissima* preparation on the first day after production reached levels of 0.50 ± 0.05 and 1.31 ± 0.03 mM TE/L, respectively. It was ca. 6- and 10-times higher in comparison to control. The application of a higher dose (0.2%) of *R. spinosissima* preparation at the same time resulted in a ca. double increase of these activities (to 0.86 ± 0.03 in DPPH and to 2.45 ± 0.02 mM TE/L in FRAP test). Moreover, the ABTS analysis showed that the application of 0.1% and 0.2% of *R. spinosissima* preparation compared to control elevated the activity 3–4 times to 2.05 ± 0.03 and to 3.18 ± 0.07 mM TE/L, respectively. The analysis points out that the dose of applied preparation was statistically significant for the antioxidant activity of yoghurts. During their cool storage, the activity did not change with time, which shows a good stability of the preparation (no significant statistical differences). The maintenance of high antioxidant activity may also be a result of the presence in the products of other bioactive compounds such as peptides and amino acids, which also function as antioxidants. Many authors in their research have shown that the antioxidant activity of yoghurts is influenced by the kind and amount of polyphenolic additive [29,34]. Analyzing yoghurts enriched with the addition of 15% and 30% of strawberry pulp, Jaster et al. [34] proved a significant elevation of antioxidant activity in those products. The determined free radical-scavenging activity measured by DPPH and ABTS was positively correlated with the amount of anthocyanins, which, however, are characterized by high instability and quick degradation. The trend was noticeable during yoghurt storage. Moreover, Raikos et al. [35], analyzing yoghurts enriched with salal berries and blackcurrant pomace, showed that storage time affected the stability of anthocyanins and antioxidant potential (as determined by FRAP) of those products. Zhang et al. [33], with both DPPH and ABTS assays, showed the stability of radical-scavenging activity in yoghurts enriched with moringa leaf extract during their refrigerated storage. Najgebauer-Lejko et al. [36], during analysis of yoghurts supplemented with pomaces (broccoli, pumpkin, red sweet pepper and carrot), observed that their antioxidant power measured as DPPH radical-scavenging ability was much more stable during storage than ferric-reducing antioxidant power, which may be due to fact that FRAP assay allows the estimation of only the water-soluble antioxidant fraction and does not take into account lipophilic substances like carotenoids, which are present in a significant amount in vegetables and also in milk. Moreover, research by Trigueros et al. [19] confirm considerable variation in antioxidant activity depending on antioxidant assay. The authors observed that pomegranate juice showed the strongest ferric-reducing capacity, whereas pomegranate yoghurt showed the highest radical-scavenging activity.

Results of the analysis of phenolic compounds in yoghurts are presented in Table 6. It shows the presence of all 10 compounds identified in the *R. spinosissima* preparation. Their level on the first day after yoghurt production was 44.71 mg/L and 82.40 mg/L in samples with 0.1% and 0.2% of *R. spinosissima* preparation supplementation, respectively. In these samples, the highest content was determined for anthocyanins, which reached the level of 19.53 and 37.38 mg/L, respectively. Among them, the highest amount was identified for cyanidin3-O-glucoside (ca. 95% of anthocyanins). The second group of polyphenols in terms of quantity were flavan-3-ols, represented by procyanidin dimer and catechin. In yoghurt enriched with 0.1% of rose preparation, their concentration was 11.27 and 7.48 mg/L, while in samples with a double dose of the additive it was 19.08 and 13.51 mg/L, respectively. Flavanols and ellagitannin concentrations in yoghurt samples compared to the mentioned two groups of polyphenols were ca. 5–6 times lower.

During storage, the amount of all phenolic compounds in yoghurt samples was not reduced, even a slight elevation was observed, especially in yoghurts enriched with a higher dose of analyzed preparation. This may be explained by the change of the protein−polyphenol interaction status, which is maximal at the isoelectric point of the protein, i.e., where yoghurt is produced [19]. According to Luck et al. [37], this interaction could be reversible or irreversible depending on pH, temperature, protein and flavonoid concentration. Our results are in contrast to the observation made by Trigueros et al. [19], who used yoghurt supplementation rich in anthocyanins pomegranate juice and observed that its main component, cyanidin-3-O-glucoside, was significantly reduced, whereas cyanidin-3,5-diglucoside was much more stable, which was explained by compound structure influence (mono or diglucoside form) on the anthocyanin stability. Moreover, Ścibisz et al. [12] observed a significant reduction of anthocyanins in yoghurt. However, the decrease in their content during storage was dependent on the fruit species. Their degradation rate was more pronounced in products with strawberry and sour cherry than with blueberry fruit. However, other authors also showed a high stability of polyphenolic compounds, including anthocyanins, in yoghurts, supplemented with mulberry extract which especially rich with these compounds [38,39]. According to Castaneda-Ovando et al. [40], stability of anthocyanins is affected by several factors such as pH, storage temperature, chemical structure, concentration, light, oxygen, solvents, the presence of enzymes, flavonoids, proteins and metallic ions. Their stabilization may be caused by intramolecular association between anthocyanins and intermolecular association with other flavonoids, amino acids, organic acids, nucleotides, polysaccharides or metals that could be present in the matrix.

## 4. Conclusions

The preparation from *R. spinosissima* is characterized by a high level of polyphenols and high antioxidant activity. It may be a valuable product used as an additive for production of functional food. Its application in yoghurt production even in minor doses of 0.1% and 0.2% did not cause radical changes in physico-chemical properties (acidity, rheological parameters) of analyzed products and also gave them a pink color. The introduced additive positively influenced the count of LAB and caused a significant elevation in antioxidant activity correlated with the preparation dose. The polyphenolic compounds, which may also be used as a natural color additive, were characterized by high stability during product storage. Finally, the use of *R. spinosissima* fruit extract was shown to be an excellent example for the production of yoghurts with pro-healthy properties.

## Figures and Tables

**Table 1:** Total polyphenols and antioxidant potential of *R. spinosissima* and purified dry extract.

Parameter	Fruit	Purified Dry Extract
Total polyphenols (mg GAE/100 g)	2643.43 ± 56.57	47,198.47 ± 226.65
DPPH (mmol TE/100 g)	11.01 ± 0.74	144.36 ± 1.87
ABTS (mmol TE/100 g)	58.87 ± 1.83	517.28 ± 3.21
FRAP (mmol TE/100 g)	18.33 ± 0.71	328.62 ± 2.86

GAE—Gallic acid equivalent, TE—Trolox equivalent, 1,1-Diphenyl-2-picrylhydrazyl radical (DPPH), 2,2′-azinobis(3-ethylbenzothiazoline-6-sulfonic acid) (ABTS), 2,4,6-tri(2-pyridyl)-s-triazine (TPTZ).

**Table 2:** Acidity changes in the yoghurt supplemented with of 0.1% and 0.2% (*w*/*v*) of *Rosa spinosissima* fruits extract.

	Day of Storage	Control	0.1%	0.2%
**pH**	1	4.38 ± 0.03 ^bcd^	4.39 ± 0.03 ^cd^	4.41 ± 0.03 ^d^
	7	4.32 ± 0.03 ^ab^	4.34 ± 0.03 ^abc^	4.35 ± 0.03 ^abcd^
	14	4.31 ± 0.03 ^a^	4.31 ± 0.04 ^a^	4.33 ± 0.04 ^abc^
**°SH**	1	42.10 ± 2.30 ^a^	42.10 ± 3.00 ^a^	44.50 ± 3.60 ^abc^
	7	46.70 ± 1.00 ^abc^	44.40 ± 4.60 ^ab^	47.40 ± 3.40 ^abc^
	14	49.60 ± 2.00 ^bc^	50.10 ± 2.00 ^b^	50.70 ± 3.50 ^c^

Letters ^a,b,c,d^ show the homogeneous groups.

**Table 3:** Color parameter (L*, a*, b*; L*: lightness; 100 = white, 0 = black, a*: redness; ±, red; green and b*: yellowness; ±, yellow; blue) changes in yoghurt during storage.

Variant	Storage Time [days]	L*	a*	b*
Control	1	86.19 ± 2.89 ^c^	−2.34 ± 0.20 ^a^	6.21 ± 0.50 ^b^
	7	88.44 ± 0.17 ^c^	−2.40 ± 0.03 ^a^	6.38 ± 0.33 ^b^
	14	88.29 ± 0.27 ^c^	−2.48 ± 0.05 ^a^	6.23 ± 0.30 ^b^
0.1%	1	72.77 ± 1.86 ^b^	8.71 ± 0.68 ^b^	−0.44 ± 0.01 ^a^
	7	74.91 ± 0.04 ^b^	8.88 ± 0.57 ^b^	−0.40 ± 0.02 ^a^
	14	73.90 ± 1.57 ^b^	8.61 ± 0.16 ^b^	−0.31 ± 0.05 ^a^
0.2%	1	69.18 ± 0.08 ^a^	13.30 ± 0.06 ^d^	−0.62 ± 0.06 ^a^
	7	68.26 ± 0.81 ^a^	12.68 ± 0.29 ^d^	−0.58 ± 0.15 ^a^
	14	68.57 ± 2.54 ^a^	11.07 ± 1.32 ^c^	−0.54 ± 0.10 ^a^

Letters ^a, b, c, d^ show the homogeneous groups.

**Table 4:** Total viable counts (log10 CFU g^−1^) in yoghurts supplemented with of 0.1% and 0.2% (*w*/*v*) of *Rosa spinosissima* fruits extract during 14 days of storage.

	Storage Time (Days)	Variant		
		Control	0.1%	0.2%
MRS	1	8.13 ± 0.12 ^cd^	8.31 ± 0.02 ^e^	8.65 ± 0.15 ^g^
	7	7.86 ± 0.05 ^b^	8.14 ± 0.06 ^cd^	8.50 ± 0.08 ^f^
	14	7.65 ± 0.07 ^a^	8.03 ± 0.05 ^c^	8.23 ± 0.04 ^de^
M17	1	8.45 ± 0.04 ^b^	8.98 ± 0.08 ^de^	9.23 ± 0.26 ^f^
	7	8.20 ± 0.10 ^a^	8.85 ± 0.07 ^cd^	9.14 ± 0.07 ^ef^
	14	8.03 ± 0.04 ^a^	8.66 ± 0.07 ^c^	8.74 ± 0.08 ^c^

Letters ^a,b,c,d,e,f,g^ show the homogeneous groups.

**Table 5:** Antioxidant activity DPPH, FRAP and ABTS (mM Trolox/L) in yoghurts with addition of 0.1% and 0.2% of *Rosa spinosissima* fruits extract.

Antioxidant Activity	Storage Time (Days)	Variant		
		Control	0.1%	0.2%
DPPH	1	0.08 ± 0.01 ^a^	0.50 ± 0.05 ^b^	0.86 ± 0.03 ^c^
	7	0.08 ± 0.02 ^a^	0.49 ± 0.03 ^b^	0.81 ± 0.04 ^c^
	14	0.07 ± 0.06 ^a^	0.47 ± 0.08 ^b^	0.86 ± 0.03 ^c^
FRAP	1	0.07 ± 0.02 ^a^	1.31 ± 0.03 ^b^	2.45 ± 0.02 ^c^
	7	0.08 ± 0.02 ^a^	1.28 ± 0.03 ^b^	2.57 ± 0.06 ^d^
	14	0.10 ± 0.06 ^a^	1.29 ± 0.01 ^b^	2.56 ± 0.08 ^d^
ABTS	1	0.70 ± 0.03 ^a^	2.05 ± 0.03 ^b^	3.18 ± 0.07 ^c^
	7	0.68 ± 0.02 ^a^	1.95 ± 0.12 ^b^	3.32 ± 0.06 ^d^
	14	0.65 ± 0.05 ^a^	2.01 ± 0.02 ^b^	3.33 ± 0.06 ^d^

Letters ^a, b, c, d^ show the homogeneous groups.

**Table 6:** Content of anthocyanins, favan-3-ols, ellagitannins and flavonols (mg/L) in yoghurts with the addition of 0.1% and 0.2% of *Rosa spinosissima* fruits extract during 14 days of storage.

Sample		Anthocyanins			Favan-3-ols			Ellagitannins			Flavonols				
Addition Rosa Ex	Stor. Day	Cy diglc	Cy glc	Total	Dimer	Cat	Total	EA	Rug	Total	Q rut	Q glc	Q p	Q der	Total
0.1%	1	1.08 ± 0.02 ^c^	18.49 ± 0.06 ^d^	19.53 _d_	11.27 ± 0.00 ^c^	7.48 ± 0.01 ^c^	18.74 ^c^	2.11 ± 0.02 ^e^	0.35 ± 0.03 ^c^	2.47 ^e^	0.07 ± 0.01 ^b^	0.21 ± 0.00 ^c^	0.05 ± 0.00 ^c^	0.07 ± 0.00 ^d^	3.97 ^c^
	7	1.08 ± 0.02 ^c^	17.82 ± 0.13 ^d^	18.79 ^cd^	11.21 ± 0.07 ^c^	7.30 ± 0.27 ^c^	18.65 ^c^	2.34 ± 0.02 ^e^	0.34 ± 0.02 ^c^	2.71 ^e^	0.07 ± 0.00 ^b^	0.21 ± 0.00 ^c^	0.05 ± 0.00 ^c^	0.07 ± 0.00 ^d^	3.94 ^c^
	14	1.11 ± 0.01 ^c^	19.20 ± 0.04 ^c^	20.30 ^c^	11.49 ± 0.08 ^c^	7.83 ± 0.23 ^c^	19.54 ^c^	1.98 ± 0.02 ^d^	0.33 ± 0.03 ^c^	2.29 ^d^	0.07 ± 0.00 ^b^	0.21 ± 0.00 ^c^	0.05 ± 0.00 ^c^	0.07 ± 0.00 ^d^	3.95 _c_
0.2%	1	1.98 ± 0.00 ^b^	35.37 ± 0.04 ^b^	37.38 ^b^	19.08 ± 0.19 ^b^	13.51 ± 0.04 ^b^	32.76 ^b^	4.41 ± 0.08 ^c^	0.54 ± 0.03 ^b^	4.88 ^c^	0.13 ± 0.01 ^a^	0.39 ± 0.01 ^b^	0.10 ± 0.00 ^b^	0.12 ± 0.00 ^c^	7.38 ^b^
	7	2.07 ± 0.05 ^b^	40.52 ± 0.09 ^a^	42.69 ^a^	21.23 ± 0.32 ^a^	16.18 ± 0.28 ^a^	37.83 ^a^	5.25 ± 0.03 ^b^	0.57 ± 0.02 ^ab^	5.85 ^b^	0.13 ± 0.01 ^a^	0.43 ± 0.00 ^a^	0.10 ± 0.00 ^b^	0.13 ± 0.00 ^b^	8.09 ^a^
	14	2.24 ± 0.11 ^a^	40.62 ± 0.68 ^a^	42.30 ^a^	21.39 ± 0.58 ^a^	16.03 ± 0.28 ^a^	36.82 ^a^	5.70 ± 0.15 a	0.61 ± 0.01 ^a^	6.20 ^a^	0.14 ± 0.00 ^a^	0.44 ± 0.01 ^a^	0.11 ± 0.00 ^a^	0.14 ± 0.00 ^a^	8.11 ^a^

**Rosa Ex**: Rosa extract; **Stor. day**: storage day; **Cy diglc**: cyanidin 3,5-*O*-diglucoside; **Cy glc**: cyanidin 3-*O*-glucoside; **Dimer**: procyanidin dimer; **Cat**: (+)-catechin; **EA:** ellagic acid; **Rug**: rugosin; **Q rut**: Quercetin 3-*O*-rutinoside; **Q glc**: Quercetin 3-*O*-glucoside; **Q p**: Quercetin 3-*O*-pentoside; **Q der**: Quercetin derivatives; Values are expressed as the mean (n = 3) standard deviation. Mean values with different letters (a, b, c, etc.) within the same column are statistically different (*p* < 0.05) and show the homogeneous groups.

## Supplementary Materials

The following are available online at https://www.mdpi.com/2304-8158/9/9/1167/s1, Figure S1. HPLC chromatograms of anthocyanins (520 nm), flavonols (360 nm), flavan-3-ols (280 nm), and ellagitanins (254 nm) of the purified rose extract. Table S1. Linear range, calibration curve, correlation coefficient R, LOD, and LOQ data for four used standards.
